# Investigating Sources of Heterogeneity in Randomized Controlled Trials of the Effects of Pharmacist Interventions on Glycemic Control in Type 2 Diabetic Patients: A Systematic Review and Meta-Analysis

**DOI:** 10.1371/journal.pone.0150999

**Published:** 2016-03-10

**Authors:** Patricia Melo Aguiar, Giselle de Carvalho Brito, Tácio de Mendonça Lima, Ana Patrícia Alves Lima Santos, Divaldo Pereira Lyra, Sílvia Storpirtis

**Affiliations:** 1 Department of Pharmacy, University of São Paulo, São Paulo, São Paulo, Brazil; 2 Department of Pharmacy, Federal University of Sergipe, São Cristóvão, Sergipe, Brazil; Hunter College, UNITED STATES

## Abstract

**Objective:**

To assess the effect of pharmacist interventions on glycemic control in type 2 diabetic patients and to examine factors that could explain the variation across studies.

**Methods:**

A comprehensive literature search was performed in PubMed, Scopus, and LILACS databases for randomized controlled trials (RCTs) published up to July 2015. The search strategy included the use of MeSH terms or text words related to pharmacist interventions, type 2 diabetes, and randomized controlled trials. RCTs published in English, Portuguese, or Spanish that evaluated the effect of pharmacist intervention on glycemic control in type 2 diabetic outpatients were included. Two independent authors executed study selection, data extraction, and risk of bias assessment. Mean differences in glycosylated hemoglobin (HbA1c) were estimated using random-effect models, and heterogeneity was evaluated by subgroup and meta-regression analyses.

**Results:**

The literature search yielded 963 records of potential interest, of which 30 were included in the systematic review and 22 in the meta-analysis. Most of these RCTs were conducted in the United States in patients in outpatient clinics using face-to-face contact only. All RCTs performed patient education, and most executed the medication review. The appraised sample showed uncertain or high risk of bias in most of the items evaluated, resulting in low-quality studies. In comparison with usual care, pharmacist interventions were associated with significant reductions in HbA1c levels (-8.5% [95% CI: -1.06, -0.65]; P < 0.0001; I^2^ = 67.3%). Subgroup analysis indicated differences of heterogeneity by country, baseline HbA1c levels, setting, intervention frequency, and random allocation. Age and HbA1c levels partly explained the variability across studies by meta-regression.

**Conclusions:**

Our findings confirmed that pharmacist interventions improve glycemic control in patients with type 2 diabetes compared with usual care and suggest that younger patients or with higher baseline HbA1c levels may be the main beneficiaries of pharmacist care.

**Protocol PROSPERO Registration Number:**

CRD42014007457

## Introduction

Type 2 diabetes is a complex chronic illness that represents a major public health problem. Its prevalence is increasing dramatically in worldwide, together with its social and economic burden of long-term complications [1]. Due to the multifaceted nature of the diabetes-related management, numerous needs and demands are requisite of the patient and healthcare providers [2]. However, there is a substantial gap between ideal and actual care, and thus, the health outcomes of patients with type 2 diabetes are often inadequate [3].

Faced with these emerging challenges, different intervention models have been developed considering the peculiarities of health systems and practice settings in order to improve the effectiveness of chronic care for diabetic patients [4,5]. A meta-analysis [6] showed that changing the structure of the primary healthcare team (i.e., including a member beyond the primary physician, such as a pharmacist, where all professionals have an active participation in management of patients) is the most effective strategy for glycemic control, principally in clinical trials that enrolled patients with mean baseline levels of glycosylated hemoglobin (HbA1c) greater than 8.0%.

Pharmacists have expanded their services further than drug dispensing, and they often participate directly in the management of patients with the delivery of clinical services [7]. Such services are considered complex health interventions as they involve multiple interacting components that, isolated or in combination, generate the power of the intervention. Pharmacist interventions can differ in a number of ways concerning the labor process, such as the type of contact with the patient, recurrence of intervention and actions taken by the pharmacist to improve the use of medicines [8]. In addition, trials can differ about the study population, the outcomes measured, or other methodological features related to the conduct of primary studies [9]. In this context, interpreting and evaluating the usefulness of the available research evidence on pharmacist interventions can be a difficult task [8].

The previous meta-analysis studies on the effect of pharmacist interventions on glycemic control in diabetic patients provide a pooled estimate based on a substantial statistical heterogeneity, usually without possible explanations for the variability across studies [10,11]. As discussed by Pigott and Shepherd [12], evaluating the effectiveness of a complex health intervention goes beyond asking whether it works. Instead, it is important to investigate how, why, when, under what circumstances, and under what conditions an intervention is more or less effective than others. In addition, studies with high heterogeneity, although they present robustness in sensitivity analyses, add little value to demonstrate the real effect of an intervention and represent a lower level of evidence [13].

In a recent overview, we revealed that there is room for further improvements in a meta-analysis for HbA1c (the reporting and methodological quality were suboptimal) and that the sources of heterogeneity is an underexplored topic [14]. Thus, attempting to explain sources of heterogeneity can be important to overcome the limitations discussed here and to guide practitioners, researchers and policymakers in the design/planning of their actions to optimize care for patients with diabetes. The study aimed to update the evidence on the effect of pharmacist interventions on glycemic control in type 2 diabetic patients and to examine factors that may explain variation among randomized controlled trials (RCTs). For this analysis of heterogeneity, the narrative summary method was used in conjunction with subgroup analysis and meta-regression.

## Methods

The protocol of this systematic review has been registered on PROSPERO 2014 (registration number: CRD42014007457). This study was conducted and reported using the following checklists: AMSTAR (Assessment of Multiple Systematic Reviews) [15] and PRISMA Statement (Preferred Reporting Items for Systematic Reviews and Meta-Analyses) [16] (S1 Appendix).

### Search strategy

A comprehensive literature search was performed in PubMed/Medline, Scopus, and Lilacs databases for randomized controlled trials (RCTs) published up to July 2015. The standardized search strategy included the use of MeSH terms or text words related to pharmacist interventions (pharmacists, pharmaceutical care, medication therapy management, pharmaceutical services); to the disease (diabetes, diabetes mellitus type 2, glycosylated hemoglobin, glycaemia, blood glucose, glycemi*); and to clinical trials (randomized controlled trial, controlled clinical trial, random allocation). The full search strategy for the PubMed/Medline database can be found in S2 Appendix. In addition, we hand-searched references of all studies for which full texts were obtained.

### Study selection

To be included in the data extraction process, the papers had to be 1) a RCT 2) published in English, Portuguese, or Spanish; 3) conducted in a community pharmacy, hospital, or primary care setting; 4) evaluated the effect of pharmacist interventions in clinical pharmacy services, defined as the activities in which the pharmacist performs a clinical decision-making process aimed at improving the patients’ health outcomes; 5) on changes in HbA1c levels of adult outpatients with type 2 diabetes. Studies that included patients with type 1 diabetes or gestational diabetes, did not define the type of diabetes investigated, or included various chronic diseases but did not report distinct outcomes for the population of interest were excluded. In addition, for studies that published the same results for HbA1c in more than one article, only the first publication was considered.

### Outcome measurement

We decided to group the data for HbA1c levels because our prior overview [14] showed that the meta-analyses evaluating this outcome had lower quality compared with another meta-analysis that pooled blood pressure and lipid profile data in patients with diabetes.

The mean changes and standard deviations (SD) were extracted as the endpoints. Missing data were computed according to the Cochrane Collaboration guidelines [13]. If required, a pharmacist intervention effect was computed from the difference between the available mean values; SD of the change were calculated from 95% confidence intervals (CI) or standard errors (SE) or P value or interquartile ranges (IQR); or if such estimations were impossible, requested from the authors. In case of no response or unavailability of data, the studies were excluded from the meta-analysis.

### Review methods

Two authors (P.M.A. and T.M.L.) independently screened the title and abstract of all tracked records to identify potentially relevant studies. The full-text articles were reviewed to determine if they met the prespecified inclusion criteria. Any disagreements were resolved by consensus through discussion.

### Data extraction

Data extraction were performed independently by two researchers (P.M.A. with G.C.B. or T.M.L.) using a spreadsheet preformatted in Microsoft Excel. For each RCT, the investigators collected the following information: country and year of publication, sample size, sex and age of subjects, duration of diabetes, description of the intervention and control groups, outcomes measures, and main conclusions.

The key components of pharmacist interventions were described based on some domains specified in the DEPICT 2 tool [17], such as 1) type of contact with recipient—how the contact with the recipient occurs and communication method; 2) setting—where the recipient received the clinical pharmacy service; 3) action(s) taken by pharmacist—what is done to address the identified problems; 4) changes in therapy—whether the pharmacist has autonomy to change prescription medication; 5) materials that support action(s)—items developed or provided by pharmacist as part of the service; and 6) frequency of contacts—number of contacts with recipient during service and duration of intervention.

All data were displayed through a systematically structured table. According to Petticrew et al. [18], this method can help the reviewer and reader to identify themes across identified studies and can facilitate the testing of prespecified theory by exploring similarities and differences among studies.

### Risk of bias assessment

The Cochrane Risk of Bias Tool [13] was used to assess the risk of bias of each included RCT. This involves judgments for random sequence generation, allocation concealment, blinding, incomplete outcome data, selective outcome reporting, and other potential sources of bias. Each item was scored as low, unclear, or high risk of bias, and the total score was obtained by assigning 1 point for each “low” answer and 0 points for “unclear” or “high” answers, for a total quality score ranging from 0 to 7. A study was considered with low quality when less than 3 of 7 items were met. Two investigators independently (P.M.A. with G.C.B. or T.M.L.) conducted this assessment, and any disagreements were resolved by consensus.

### Data synthesis and analyses

The quantitative synthesis of HbA1c levels was conducted in the form of a meta-analysis. Mean differences and their corresponding 95% confidence intervals were used to pool intervention effect estimates. Heterogeneity across studies was examined using the Q-test (i.e., assesses whether observed differences in results are compatible with chance alone, with a P value of 0.10 as a cut-off for significance) and I-squared statistic (i.e., percentage of variation due to heterogeneity rather than chance, with values over 50% showing a substantial level of heterogeneity) [13]. Given the heterogeneity across studies, we decided to use the random-effects meta-analysis model of DerSimonian and Laird.

In addition, subgroup analysis and univariate meta-regression for categorical and continuous covariates, respectively, were performed to explore heterogeneity among the primary studies. These covariates were a priori specified and described according to the framework proposed by Pigott et al. [12] for complex intervention: substantive features related to the context (geographical setting); to the target participants (sex and baseline HbA1c level); to the intervention (key components of pharmacist interventions); and to particulars of the methods and procedures used to conduct the study (random sequence generation).

Two sensitivity analyses verified whether the exclusion of low-quality studies (risk of bias total score < 3 points) and clinical trials with sample size smaller than 100 patients influenced the results. Publication bias was measured by a visual inspection the symmetry of funnel plot and Egger's regression test. The results of the meta-analysis and meta-regression were presented as a forest plot and bubble plot, respectively, with squares and circles representing the estimates from each study, sized according to the precision of each estimate. All analyses were executed using RStudio program 0.99.473 (RStudio, Boston, MA) with the packages “Meta” 4.3–0 (General Package for Meta-analysis) and “Metaphor” 1.9–7 (Meta-analysis Package for R).

## Results

### Search results

**Fig 1:**
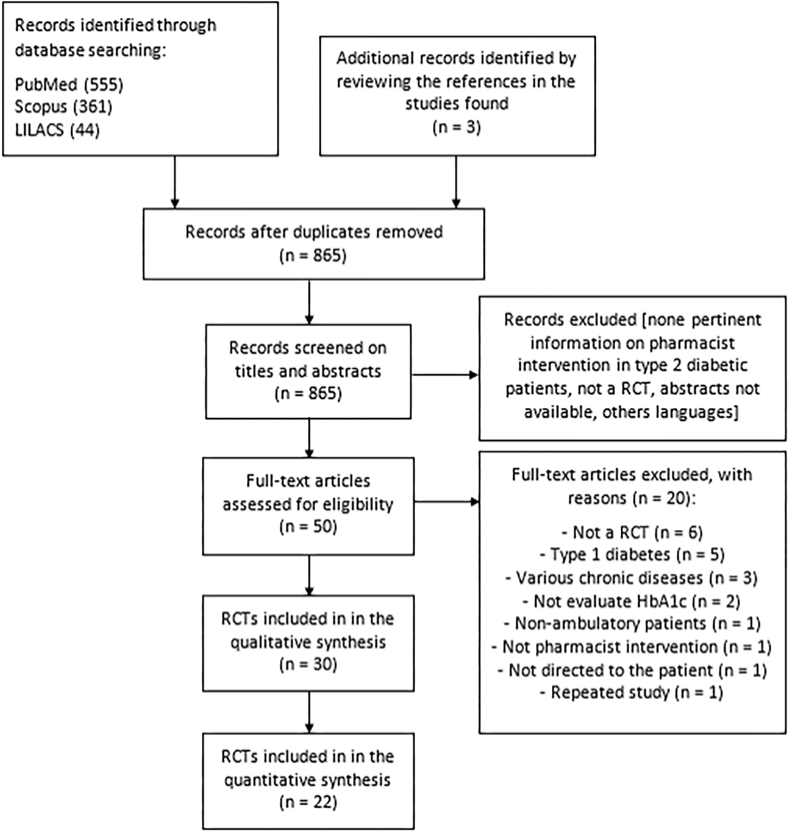
Study selection flowchart through literature search.

The reviewers initially identified 963 records from the databases, of which 30 RCTs met our inclusion criteria [19–48]. A flowchart of the selection process of studies according to inclusion and exclusion criteria is shown in Fig 1. Excluded studies after reading the full text and reasons are listed in S3 Appendix. The interobserver agreement was high for the screening process (kappa = 0.898 [95% CI: 0.831, 0.964] for the title plus abstract; and 0.834 [95% CI: 0.557, 1.0] for full text).

### Characteristics of primary studies

The characteristics of the 30 RCTs included in the systematic review are showed in Table 1. The majority of studies were conducted in North America (n = 13, with 12 in the United States). Sample size ranged from 39 to 360 patients, and approximately two-thirds of the RCTs had sample sizes >100. Most of the trials had a mean age ≥60 years (16/29, 56.6%), and higher proportion of women (15/29, 51.7%). Just over half of the trials (17/30, 56.7%) reported the diabetes duration, and of these, 76.5% of the patients had been diagnosed with this disease for <10 years. Approximately two-thirds of the RCTs had baseline HbA1c levels <9%.

**Table 1:** Description of each included RCT: country, population, components of pharmacist intervention, description of control group and outcomes measures.

Authors, year; country	Population*: mean age; % male; DM duration; HbA1c (%)	Key components of pharmacist interventions**	Description of control group	Outcome measures
Jaber et al., 1996 [19]; USA	n = 39 /17, 22; mean age: 59±12,65±12; % male: 29.4%, 31.8%; DM duration: 6.8±4.8, 6.2±4.8; HbA1c: 11.5±2.9, 12.2±3.5	1) Individual face-to-face contact; 2) University-affiliated internal medicine outpatient clinic; 3) Evaluated and adjusted hypoglycemic regimen; provided diabetes education, medication counseling, and instructions on diet, exercise and self-monitoring; 4) Autonomy to change prescription medication ***; 5) Written instructions and diary for self-monitoring of blood glucose; 6) Weekly until glycemic control was reached; thereafter, every 2–4 weeks / 4 months	Received standard care from primary care physician (usually every 3–4 months)	HbA1c; fasting blood glucose; BP; lipid levels; serum creatinine; microalbumin; QoL
Guirguis et al., 2001 [20]; Canada	n = 49 / 26, 23; mean age: 57.1±12.4, 61.9±9.4; % male: 50%, 57%; DM duration: 7.4±7.3, 6.3±5.8; HbA1c: 7.9, 7.9	1) Individual face-to-face contact; 2) Community pharmacies; 3) Provided diabetes education; medication counseling; instructions on diet, exercise and self-monitoring; and performed medication review and suggested to the physician changes in pharmacotherapy; 4) No autonomy to change prescription medication; 5) Not provided support resources; 6) Two visits in the first month; thereafter, approximately once a month / 6 months	Usual care provided by community pharmacies (can vary from medication dispensing and counselling to disease management)	HbA1c; attitudes toward diabetes; diabetes self-care activities; diabetes lifestyle; satisfaction; QoL
Sarkadi et al., 2004 [21]; Sweden	n = 71 / 33, 38; mean age: 66.4±7.9, 66.5±10.7; % male: NR; DM duration: 5.9±5.8, 2.6±2.2; HbA1c: 6.4, 6.5	1) Group face-to-face contact; 2) Community pharmacies; 3) Provided diabetes educational program (self-management, nutritional components, exercise and support for dealing with the emotional aspects) and referred to the physician when glucose control seemed unsatisfactory despite adequate diet and exercise; 4) Not applicable; 5) Video on how to “live well” with diabetes; dice game; booklet or guide on “how to manage your diabetes” and diary about learning experience; 6) Once a month/ 12 months	NR	HbA1c
Choe et al., 2005 [22]; USA	n = 80 / 41, 39; mean age: 52.2±11.2, 51.0±9.0; % male: 48.8%, 46.1%; DM duration: NR; HbA1c: 10.1±1.8, 10.2±1.7	1) Individual face-to-face contact plus remote contact; 2) University-affiliated ambulatory care clinic; 3) Evaluated and adjusted therapeutic regimen; and patient education about diabetes self-management skills (self-care, medications, and screening processes) and sent condensed diabetes status updates to physician; 4) Autonomy to change prescription medication ***; 5) Not provided support resources; 6) In conjunction with routine primary care visits plus monthly telephone contacts / 12 months	Received regular follow-up visits with primary care physicians	HbA1c
Clifford et al., 2005 [23]; Australia	n = 180 / 92, 88; mean age: 70.5±7.1, 70.3±8.3; % male: 47.8%, 56.8%; DM duration:10.0 [range 7.6–14.0], 8.0 [range 6.6–12.0]; HbA1c: 7.5 [range 6.9–8.1], 7.1 [range 6.3–7.8]	1) Individual face-to-face contact plus remote contact; 2) Community-based diabetes center; 3) Provided patient counseling about diet, exercise, self-monitoring and medication adherence; performed medication management, suggested changes in pharmacotherapy, and delivered monitoring results report to the physician; 4) No autonomy to change prescription medication; 5) Educational pamphlets; and patient’s medication list to the physician; 6) At baseline, 6 and 12 months plus 6-weekly telephone contacts / 12 months	Standard care that included review at 6 and 12 months of blood pressure, fasting biochemical tests and lifestyle issue reinforcement	HbA1c; fasting blood glucose; BP; lipid levels; urinary albumin-creatinine ratio; BMI
Odegard et al., 2005 [24]; USA	n = 77 / 43, 34; mean age: 51.6±11.6, 51.9±10.4; % male: 52%, 62%; DM duration: 6.9±5.3, 8.3±7.5; HbA1c: 10.2±0.8, 10.6±1.4	1) Individual face-to-face contact plus remote contact; 2) University-affiliated neighborhood clinics; 3) Provided diabetes education based on individual needs; referral to other professionals (nutrition counseling and ophthalmology evaluation); and performed medication management and suggested to the physician changes in pharmacotherapy; 4) No autonomy to change prescription medication; 5) Written materials from standard sources (eg, ADA); 6) Weekly or monthly, based on diabetes care needs / 6 months	Continued normal care with their primary care provider	HbA1c; medication adherence; appropriateness of therapy
Rothman et al., 2005 [25]; USA	n = 217 / 112, 105; mean age: 54±13, 57±11; % male: 44%, 44%; DM duration: 8±9, 9±9; HbA1c: 11±2, 11±3	1) Individual face-to-face contact plus remote contact; 2) Academic general internal medicine clinic; 3) Evaluated and adjusted therapeutic regimen; and patient education and counseling about diabetes and medications;4) Autonomy to change prescription medication ***; 5) Not provided support resources; 6) Every 2–4 weeks or more frequently if indicated / 12 months	Received usual care from primary care provider	HbA1c; BP; weight; total cholesterol; use of clinical services; adverse events; diabetes knowledge; satisfaction
Suppapitiporn et al., 2005 [26]; Thailand	n = 360 / 180, 180; mean age: 61.4±10.6, 59.9±11.5; % male: 32.9%, 35.6%; DM duration: NR; HbA1c: 8.2±1.4, 8.0±1.5	1) Individual face-to-face contact; 2) Hospital-based outpatient clinic; 3) Patient education and counseling about diabetes and medications for all 4 groups; 4) Not applicable; 5) One group received information booklet, other received special medication container and other received both materials; 6) At baseline, 3 and 6 months / 6 months	NR	HbA1c; fasting blood glucose
Fornos et al., 2006 [27]; Spain	n = 112 / 56, 56; mean age: 62.4±10.5, 64.9±10.9; % male: 42.9%, 42.9%; DM duration: NR; HbA1c: 8.4±1.8, 7.8±1.7	1) Individual face-to-face contact; 2) Community pharmacies; 3) Provided patient education about diabetes, lifestyle and medications; and performed medication management and suggested to the physician changes in pharmacotherapy; 4) No autonomy to change prescription medication; 5) Written information about medication; 6) Monthly interview / 13 months	Received usual care	HbA1c; fasting blood glucose; BP; lipid levels; albumin-creatinine ratio; diabetes knowledge
Scott et al., 2006 [28]; USA	n = 149 / 76, 73; mean age: NR; % male: 42.1%, 35.6%; DM duration: NR; HbA1c: 8.8, 8.7	1) Individual and group face-to-face contact plus remote contact; 2) Community health center; 3) Provided patient education about diabetes, lifestyle, self-monitoring and adherence medication; referred to other professionals (eye or dental care); and performed medication management and suggested to the physician changes in pharmacotherapy; 4) No autonomy to change prescription medication; 5) A free blood glucose monitor and test strips; 6) Two-week follow-up sessions in initial three months; thereafter, at 3, 6 and 9 months / 9 months	Received standard care (patient education and monitoring blood glucose levels) and were managed by a nurse. Received a free blood glucose monitor plus test strips	HbA1c; BP; weight; BMI; LDL cholesterol; HDL cholesterol; QoL
Krass et al., 2007 [29]; Australia	n = 289 / 149, 140; mean age: 62±11; % male: 51%; DM duration: 8.6, 10.4; HbA1c: 8.9±1.4, 8.3±1.3	1) Individual face-to-face contact; 2) Community pharmacies; 3) Provided patient education about diabetes, self-monitoring, lifestyle and medication; delivered monitoring results report to the patient; and referred to the physician to changes in pharmacotherapy; 4) No autonomy to change prescription medication; 5) A free blood glucose monitor; 6) Five times over 6 months	Received usual care (i.e. no specialized diabetes service in the pharmacy)	HbA1c; BMI; BP; total cholesterol; triglycerides; QoL
Al Mazroui et al., 2009 [30]; United Arab Emirates	n = 240 / 120, 120; mean age: 48.7±8.2, 49.9±8.3; % male: 70%, 68.3%; DM duration: 6.1±2.9, 6.2±2.7; HbA1c: 8.5[95% CI: 8.3–8.7], 8.4[95% CI: 8.2–8.6]	1) Individual face-to-face contact; 2) Hospital-based endocrinology and medical clinic; 3) Provided patient education about diabetes, lifestyle, self-monitoring, medication and, smoking cessation; performed medication management and suggested to the physician changes in pharmacotherapy; 4) No autonomy to change prescription medication; 5) Educational leaflets and self-monitoring record book;6) At baseline, 4, 8 and 12 months / 12 months	Received normal care from medical and nursing staff (e.g. advice on self-monitoring blood glucose)	HbA1c; fasting blood glucose; BP; lipid levels; BMI; CDH risk; QoL
Doucette et al., 2009 [31]; USA	n = 78 / 36, 42; mean age: 58.7±13.3, 61.2±10.9; % male: 38.2%, 46.3%; DM duration: NR; HbA1c: 8.0±1.5,7.9±1.9	1) Individual face-to-face contact; 2) Community pharmacies; 3) Provided patient counseling about self-care activities and clinical goals; performed medication management, suggested changes in pharmacotherapy and sent a progress note to patient’ physician; 4) No autonomy to change prescription medication; 5) A medication list; 6) Four quarterly visits / 12 months	Received usual diabetes care from primary care provider	HbA1c; BP; LDL cholesterol; diet, exercise, and diabetes self-care activities
Jamenson et al., 2010 [32]; USA	n = 103 / 52, 51; mean age: 49.3±10.8, 49.7±10.9; % male: 48.9%, 49.0%; DM duration: NR; HbA1c: 10.4±1.2, 11.1±1.6	1) Individual face-to-face contact plus remote contact; 2) Community-based primary care setting; 3) Evaluated and adjusted therapeutic regimen; and patient education about diet, exercise, self-monitoring, medications, and insulin; and assessed adherence and barriers to optimizing blood glucose levels; 4) Autonomy to change prescription medication ***; 5) Not provided support resources; 6) Visits was based on the need to educate the patient about diabetes control or to monitor therapeutic changes / 12 months	Received targeted patient outreach	HbA1c
Taveira et al., 2010 [33]; USA	n = 109 / 58, 51;mean age: 62.2±10.3, 66.8±10.2;% male: 91.4%, 100%;DM duration: NR;HbA1c: 7.9±1.1, 8.1±1.5	1) Group face-to-face contact; 2) Veterans Affairs medical center; 3) Patient education about disease, diet and exercise; counseling about diabetes self-care behaviors; and evaluated and adjusted therapeutic regimen; 4) Autonomy to change prescription medication ***; 5) An individualized report card containing medical history, medications, vital signs, and laboratory test values; and handouts of tobacco cessation; 6) Once a week / 1 month	Received standard care from primary care provider (usually every 4 months)	HbA1c; BP; LDL cholesterol; non-HDL cholesterol
Cohen et al., 2011 [34]; USA	n = 99 / 50, 49; mean age: 69.8±10.7, 67.2±9.4; % male: 100%, 96%; DM duration: NR; HbA1c: 7.8±1.0, 8.1±1.4	1) Group face-to-face contact; 2) Veterans Affairs medical center;3) Patient education about disease, diet and exercise; counseling about diabetes self-care behaviors; and evaluated and adjusted therapeutic regimen; 4) Autonomy to change prescription medication ***; 5) An individualized report card containing medical history, medications, vital signs, and laboratory test values; and educational presentation slides; 6) Four once-weekly sessions followed by 5 monthly sessions / 6 months	Received standard care from primary care provider (usually every 4 months)	HbA1c; systolic BP; weight; LDL cholesterol; diabetes self-care activities; QoL; medication adherence
Farsaei et al., 2011 [35]; Iran	n = 172 / 86, 86; mean age: 53.4±9.8, 52.9±8.5; % male: 36.8%, 31.8%; DM duration:10.8±5.3, 10.3±8.2; HbA1c: 9.3±1.7, 8.9±1.1	1) Individual face-to-face contact plus remote contact; 2) Endocrine and metabolism research center; 3) Provided patient education about hypoglycemic; medications, medication adherence, diet, exercise and use of medications in the holy month of Ramadan; 4) Not applicable; 5) Diabetes diary log, pill box and medication schedule; 6) Weekly telephone contacts and appointments / 3 months	Received the general education offered by the nursing staff	HbA1c; fasting blood glucose
Mehuys et al., 2011 [36]; Belgium	n = 288 / 153, 135; mean age: 63.0 [range 40–84], 62.3[range 45–79]; % male: 51.0%, 53.7%; DM duration: NR; HbA1c: 7.7±1.7, 7.3±1.2	1) Individual face-to-face contact; 2) Community pharmacies; 3) Provided patient education about diabetes, lifestyle, hypoglycemic medications, medication adherence and reminders about annual eye and foot examinations; 4) Not applicable; 5) A free blood glucose monitor and diabetes self-management diary; 6) At each prescription-refill visit for hypoglycemic medication / 6 months	Received usual pharmacist care	HbA1c; fasting blood glucose; medication adherence; diabetes knowledge; diabetes self-care activities
Sriram et al., 2011 [37]; India	n = 120 / 60, 60; mean age: 53.6±2.4, 58.0±2.6; % male: 50.0%, 50.0%; DM duration: NR; HbA1c: 8.44±0.29, 9.03±0.46	1) Individual face-to-face contact plus remote contact; 2) Hospital-based general medicine clinic; 3) Provided medication counseling, instructions on dietary regulation, exercise and others lifestyles modifications; 4) Not applicable; 5) Information leaflet, diabetic diet chart and diabetic diary; 6) Every 3 months / 8 months	Did not receive any pharmaceutical care	HbA1c; fasting blood glucose; QoL; BMI; diabetes treatment satisfaction
Taveira et al., 2011 [38]; USA	n = 88 / 44, 44; mean age: 60.2±9.3, 61.4±9.9; % male: 100%, 95.5%; DM duration: 9.5±10.1, 9.3±7.4; HbA1c: 8.3±1.7, 8.5±1.9	1) Group face-to-face contact; 2) Veterans Affairs medical center; 3) Patient education about disease, diet and exercise; counseling about diabetes self-care behaviors; and evaluated and adjusted therapeutic regimen;4) Autonomy to change prescription medication ***; 5) An individualized report card containing medical history, medications, vital signs, and laboratory test values; educational handouts and presentation slides; 6) Four once-weekly sessions followed by 5 monthly sessions / 6 months	Received standard care from primary care provider plus 4 once weekly educational visits provided by pharmacists, nutritionists, and nurses.	HbA1c; systolic BP; LDL cholesterol, non-HDL cholesterol; CDH risk; diabetes self-care activities; self-perceived competence; depressive symptoms
Ali et al., 2012 [39]; United Kingdom	n = 46 / 23, 23; mean age: 66.4±12.7, 66.8±10.2; % male: 43.5%, 56.5%; DM duration: 7.5±4.8, 6.8±3.5; HbA1c: 8.2±1.7, 8.1±1.0	1) Individual face-to-face contact; 2) Community pharmacies; 3) Performed medication review with emphasis on adherence and identification of adverse effects; provided patient counseling about lifestyle modification and referred to a general practitioner or other healthcare professional; 4) No autonomy to change prescription medication; 5) A diabetes record book; 6) Every month for the first 2 months, and then every 3 months for the remainder / 12 months	Usual care provided by general practitioner, practice nurse and community pharmacy	HbA1c; fasting blood glucose; BP; lipid levels; BMI; QoL; diabetes knowledge; satisfaction; believes about medicines; hypoglycemic ⁄ hyperglycemic episodes
Chan et al., 2012 [40]; Hong Kong	n = 105 / 51, 54; mean age: 63.2±9.5, 61.7±11.2; % male: 58.8%, 51.9%; DM duration: 14.9±5.6, 13.8±6.8; HbA1c: 9.7±1.4, 9.5±1.8	1) Individual face-to-face contact; 2) Hospital-based diabetes clinic; 3) Provided patient education about cardiovascular diseases and lifestyle; counseling about medication (adherence, side effects, administration); performed medication review and notified the physicians for any identified drug-related problem; updated of patient's medication list; 4) No autonomy to change prescription medication; 5) Written educational materials; and color stickers for pillboxes or drug bags; 6) Before each physician visit / 9 months	Received the same medical care without pharmacist interventions	HbA1c; BP; lipid levels; BMI; urinary albumin-creatinine ratio; CHD risk; cost-effectiveness; medication adherence
Jacobs et al., 2012 [41]; USA	n = 164 / 72, 92; mean age: 62.7±10.8, 63.0±11.2; % male: 68%, 55%; DM duration: NR; HbA1c: 9.5±1.1, 9.2±1.0	1) Individual face-to-face contact; 2) Ambulatory general internal medicine; 3) Provided patient education about diabetes; counseling about diet, exercise, medications and self-monitoring; referral to other clinicians (e.g. ophthalmologist); and performed medication management and suggested to the physician changes in pharmacotherapy; 4) No autonomy to change prescription medication; 5) Not provided support resources; 6) At baseline, 6, and 12 months; if necessary, additional visits were scheduled / 12 months	Received usual care directed by physician	HbA1c; BP; LDL cholesterol
Jarab et al., 2012 [42]; Jordan	n = 171 / 85, 86; mean age: 63.4±10.1, 65.3±9.2; % male: 57.6%, 55.8%; DM duration: 9.7±7.4, 10.1±7.7; HbA1c: 8.5 [range 6.9–10.3], 8.4[range 6.6–10.2]	1) Individual face-to-face contact plus remote contact; 2) Hospital-based diabetes clinic; 3) Provided patient education about diabetes, prescribed drug therapy, medication adherence and lifestyle; referred to a special smoking cessation program run within the hospital when necessary; and performed medication management and suggested to the physician changes in pharmacotherapy; 4) No autonomy to change prescription medication; 5) A special booklet on diabetes medications and lifestyle; 6) At baseline and 6 months; followed by 8-weekly telephone follow-up / 6 months	Usual care provided by the medical and nursing staff (review for blood glucose and BP, advice on self-monitoring of blood glucose and nutrition counseling)	HbA1c; fasting blood glucose; BP; lipid levels; BMI; diabetes self-care activities; medication adherence
Mourão et al., 2012 [43]; Brazil	n = 100 / 50, 50; mean age: 60.0±10.2, 61.3±9.9; % male: 32.0%, 34.0%; DM duration: NR; HbA1c: 9.9±2.1, 9.5±1.8	1) Individual face-to-face contact; 2) Primary health care units; 3) Provided patient education about diabetes, non-pharmacological issues and pharmacological treatments; and performed medication management and suggested to the physician changes in pharmacotherapy; 4) No autonomy to change prescription medication; 5) Not provided support resources; 6) Once a month / 6 months	Received usual health care provided by doctor, nurse, nutritionist or physiotherapist	HbA1c; fasting blood glucose; BP; lipid levels; BMI
Castejon et al., 2013 [44]; USA	n = 43 / 19, 24; mean age: 54.0±9.0, 55.0±10.0; % male: 42.0%, 21.0%; DM duration: NR; HbA1c: 8.3±0.4, 8.2±0.4	1) Individual and group face-to-face contact; 2) Community setting in partnership with a community-based organization; 3) Provided diabetes education, nutrition, exercise, self- monitoring of blood glucose; and performed medication therapy management; 4) No autonomy to change prescription medication; 5) Animated video titled, ‘What is Diabetes/¿Qué es la Diabetes?’ by Animax Health, 2006 Health LAMP, Inc.; and monitors and strips; 6) Every two weeks during the first six weeks and a follow- up three months later / four and a half months	The control group had the same timeline but no educational sessions were given	HbA1c; random blood glucose; BP; lipid levels; weight; BMI; waist circumference
Chung et al., 2014 [45]; Malasya	n = 241 / 120, 121; mean age: 59.7±9.5, 58.5±8.3; % male: 41.7%, 46.3%; DM duration: 16.3±8.0, 16.3±8.0; HbA1c: 9.6±1.3, 9.5±1.4	1) Individual face-to-face contact plus remote contact; 2) University-affiliated diabetes clinic; 3) Provided educated on diabetes, hypertension, and hyperlipidemia; instructions about medications and medication adherence; and performed medication review; 4) No autonomy to change prescription medication;5) Pill box and blood glucose meter; 6) Monthly follow-up telephone and visits every 3–4 months/12 months	Provided standard pharmacy services, which consisted of dispensing the medications and providing brief instructions on how to take them	HbA1c; fasting blood glucose; medication adherence
Wishah et al., 2014 [46]; Jordan	n = 106 / 52, 54; mean age: 52.9±9.6, 53.2±11.2; % male: 38.5%, 48.1%; DM duration: 5.5±4.5, 5.1±4.9; HbA1c: 8.9±1.6, 8.2±1.3	1) Individual face-to-face contact plus remote contact; 2) University-affiliated outpatient diabetes clinic; 3) Provided patient education and counseling about diabetes, risks for diabetes complications, prescribed medications, proper dosage, possible side effects, and importance of adherence to diabetes self-care activities; performed medication management and sent recommendation for physician about changes in pharmacotherapy; 4) No autonomy to change prescription medication; 5) Printed educational leaflet and brochures containing information about diabetes, diabetes medications, life-style modifications, and self-care activities; 6) Every 1–3 months, depending on the glycemic control for each patient /6 months	Regular follow-up clinic visits every 1–3 months, depending on the glycemic control for each patient. Provided by the medical and nursing staff	HbA1c; fasting blood glucose; lipid levels; BMI; BP; knowledge about diabetes; medication adherence; diabetes self-care activities
Cani et al., 2015 [47]; Brazil	n = 70 / 34, 36; mean age: 61.9±9.6, 61.6±8.1; % male: 38.2%, 38.9%; DM duration: 14.6±7.4, 14.9±8.5; HbA1c: 9.78±1.55, 9.61±1.38	1) Individual face-to-face contact; 2) University-affiliated diabetes outpatient clinic; 3) Provided patient education about diabetes, acute and chronic complications, lifestyle (diet, physical activity, smoking cessation), regular foot inspections and blood glucose monitoring; patient counseling on indication, proper dosage, side effects and adequate storage of medication; performed medication review and sent recommendations to physician, such as insulin dose adjustments; 4) No autonomy to change prescription medication; 5) Pill organizers and written guidance on prescriptions; 6) Monthly/ 6 months	Received standard care	HbA1c; diabetes and medication knowledge; QoL; medication adherence; insulin injection and home blood glucose monitoring techniques
Jahangard-Rafsanjani et al., 2015 [48]; Iran	n = 85 / 45, 40; mean age: 57.3±8.6, 55.9±8.7; % male: 44.4%, 35.0%; DM duration: 4.6±4.3, 5.7±5.9; HbA1c: 7.6±1.6, 7.51±1.9	1) Individual face-to-face contact plus remote contact; 2) Community pharmacy; 3) Provided patient education on diet management, physical activity and diabetes complications; counseling on self-monitoring and medication adherence; performed medication review and referred to the physician whenever a drug therapy modification was required; 4) No autonomy to change prescription medication; 5) Blood glucose self-monitoring device and test strips, special logbook and educational pamphlets for the diabetes medications; 6) Five follow-up visits (once a month) plus telephone call between visits / 5 months	Received usual care from the physician. The end of the study the community pharmacist provided a brief education on diabetes self-care	HbA1c; BP; medication adherence; diabetes self-care activity; BMI; satisfaction

* Population reported as intervention group, control group. If not available, the data of the total population will be presented.

** Description based on the DEPICT tool: 1) Type of contact with the patient; 2) Setting; 3) Action taken by pharmacist to address the identified problems; 4) Pharmacist’s autonomy to change prescription medication; 5) Support resources provided by pharmacist; and 6) Frequency /duration of intervention.

*** With restrictions.

RCT—randomized controlled trial; USA—United States of America; DM—diabetes mellitus; HbA1c—glycosylated hemoglobin; BP—blood pressure; QoL—quality of life; BMI—body mass index; CDH—coronary heart disease; NR—not reported.

The assessment of the key components of pharmacist interventions showed that half of the trials were conducted in outpatient clinics of a hospital (50.0%); the contact with the patient often occurred individually (80.0%); and main communication method was face-to-face contact only (60.0%). The duration of the pharmacist interventions ranged from 1 to 13 months, with an average of 8.1 months. In addition, in half of the included studies the pharmacist had established regular (at least once a month) contact with the patient.

Most studies performed the medication review (25, 83.3%), and different terms to indicate this service were used, with “pharmaceutical care” the most frequent (10/25, 40.0%). In addition, most of the studies (15/25, 60%) did not describe clearly how the medication review process was performed, and when the term "drug-related problems” or similar was used, there was no explanation or reference to the classification of such problems (8/10, 80.0%).

Pharmacists have taken multifaceted actions to address the identified problems in the care of diabetic patients, including educating patients (concerning diabetes, lifestyle, and self-monitoring) or providing medication counseling (100.0%); sending suggestions or recommendations to the physician regarding changes in medication (46.7%); adjusting pharmacotherapy on the basis of protocols previously established in collaboration with the healthcare team (23.3%); and referring patients to other heath professional (e.g., dentist) (16.7%). To support their actions, pharmacists frequently provided educational material (50.0%) and diabetes self-management diaries (23.3%) to their patients.

In general, the control group received usual care, although some were also given access to educational programs or a free blood glucose monitor. Usual care was defined differently among the studies as the normally received care from a general practitioner and sometimes from other healthcare professionals (e.g., nurses). In addition to HbA1c values, the clinical outcomes measured most frequently were blood pressure (63.3%), low-density lipoprotein cholesterol (53.3%), and fasting blood glucose (46.7%); quality of life was evaluated as the main humanistic outcome (33.3%), whereas diabetes self-care activities were measured most frequently as a process indicator (26.7%).

### Risk of bias

The studies showed a variable methodological quality based on assessment using the Cochrane Risk of Bias Tool (S4 Appendix). The allocation sequence was adequately reported in 53.3% of the studies (16/30), and random number tables or a computer-generated randomized list were the most commonly used methods. Most RCTs (25/30) did not describe at all or did not described in sufficient detail the allocation concealment to allow a judgment. None of the trials blinded participants and personnel to pharmacist intervention, and only four of them reported that the outcome assessment was executed by an assessor blinded to treatment assignment.

Half of the studies were unclear or had some incomplete data for HbA1c (e.g., no reasons provided for missing data or the reason for missing outcome data was likely related to the HbA1c test with imbalances across groups). Only one study indicated the existence of a protocol and all pre-specified outcomes have been reported; in addition, the majority of trials provided insufficient information to permit judgment. Less than half of the analyzed studies (12/30) were not free of other bias (e.g., baseline imbalance in population characteristics). Thus, only 12 trials met the criterion of a quality score of at least 3 and could be classified as good quality.

### Meta-analysis for HbA1c

**Fig 2:**
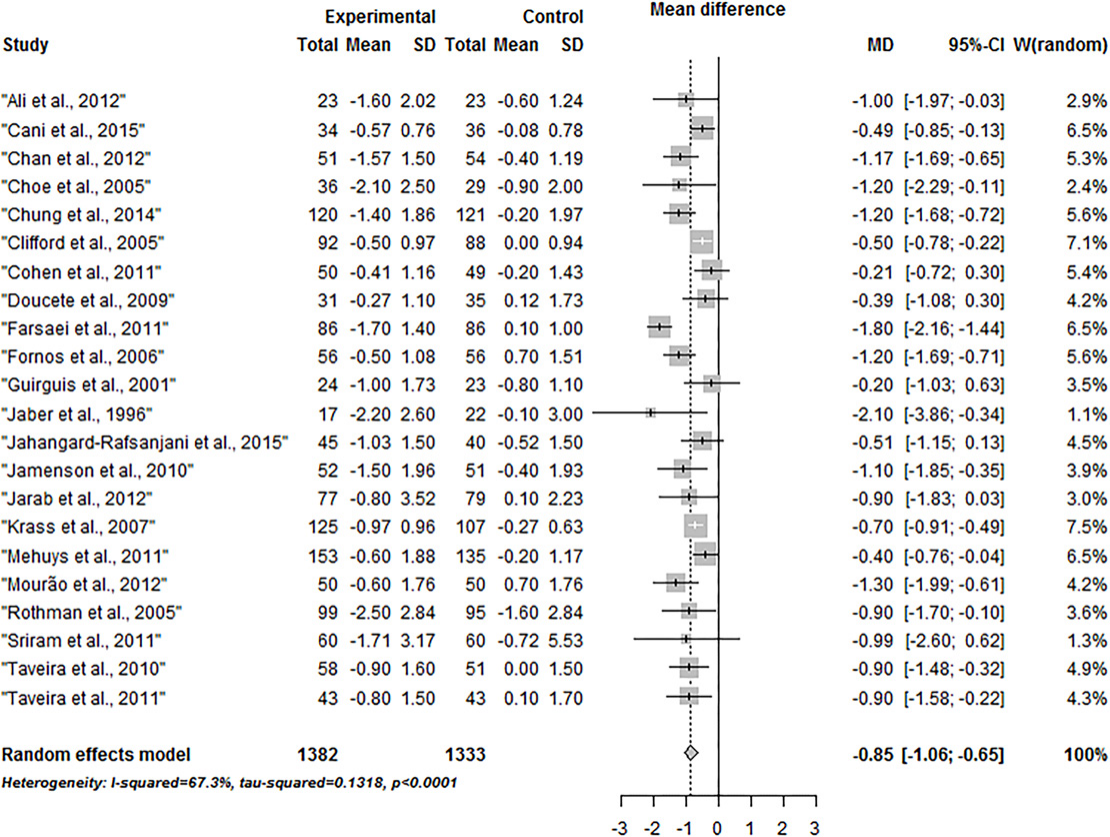
Forest plot of the mean difference of HbA1c in the pharmacist intervention compared with usual care group.

Of 30 RCTs from the systematic review, only 20 adequately reported the outcome of interest. Therefore, the authors of the other trials were contacted to obtain additional unpublished data; two of them sent information that allowed calculation of the missing data. Finally, 22 studies were included in this meta-analysis on the effect of pharmacist health interventions in glycemic control in patients with type 2 diabetes. The pooled effect estimate was significant (see Fig 2), with a uniform direction of effect in favor of pharmacist intervention (mean difference for HbA1c of -0.85% [95% CI: -1.06, -0.65]; P < 0.0001); heterogeneity was significant (P < 0.0001) and considerable (I^2^ = 67.3%). To explore this heterogeneity across included studies, we performed subgroup analysis and meta-regression.

### Subgroup and meta-regression analyses

Subgroup analysis identified no significant difference in the reduction in HbA1c levels according to the country where the study was conducted, type of contact with the patient, whether a medication review was performed; autonomy of the pharmacist to change drug therapy, provision of support resources, frequency of intervention, and adequate random allocation (Table 2). The effect on HbA1c levels tended to be more important if the pharmacist interventions were conducted in other outpatient settings compared with a community pharmacy, although the analysis identified no significant difference (-0.98% vs. -0.65%; P = 0.08). The main result favoring the intervention group were seen when these trials involved patients with mean baseline HbA1c values > 9.0% compared with values ≤ 9% (-1.18% vs. -0.63%; P = 0.007).

**Table 2:** Subgroup Analyses for the mean difference of HbA1c in the pharmacist intervention compared with usual care group.

Covariates	Number of trials	Mean difference [95% CI]	P value between groups	Heterogeneity I^2^ (P value)
*Country*			0.594	
Others	14	-0.88 [-1.13, -0.62]		76.1% (P < 0.001)
United States	8	-0.77 [-1.07, -0.46]		26.9% (P = 0.214)
*Baseline HbA1c levels*			0.007	
≤ 9%	13	-0.63 [-0.78, -0.47]		19.3% (P = 0.249)
> 9%	9	-1.18 [-1.55, -0.81]		70.2% (P < 0.001)
*Contact type*			0.234	
Only face-to-face	13	-0.73 [-0.9, -0.53]		48.7% (P = 0.025)
Plus remote contact	9	-1.02 [-1.44, -0.60]		76.5% (P < 0.001)
*Community pharmacy setting*			0.079	
No	15	-0.98 [-1.26, -0.69]		71.7% (P < 0.001)
Yes	7	-0.65 [-0.88, -0.41]		36.0% (P = 0.153)
*Medication review*			0.625	
No	3	-1.07 [-2.22, 0.07]		93.1% (P < 0.001)
Yes	19	-0.79 [-0.95, -0.60]		38.0% (P = 0.048)
*Support material*			0.614	
No	5	-0.95 [-1.33, -0.57]		11.7% (P = 0.339)
Yes	17	-0.83 [-1.07, -0.60]		72.8% (P < 0.001)
*Autonomy to medication change*			0.949	
No	15	-0.85 [-1.10, -0.60]		74.8% (P < 0.001)
Yes	7	-0.83 [-1.17, -0.49]		29.6% (P = 0.202)
*Intervention frequency*			0.141	
≤ 1 month	13	-0.94 [-1.26, -0.62]		73.4% (P < 0.001)
> 1 month or not reported	9	-0.67 [-0.84, -0.49]		19.4% (P = 0.270)
*Adequate random allocation*			0.375	
No	10	-0.74 [-1.07, -0.41]		82.2% (P < 0.001)
Yes	12	-0.92 [-1.11, -0.72]		7.8% (P = 0.369)

Furthermore, we observed statistical heterogeneity unimportant (0% to 40%) and non-significant (P > 0.10) in the studies with the following characteristics: conducted in the United States; patients with HbA1c baseline levels ≤ 9%; conducted in a community pharmacy; no support material provided by pharmacist; pharmacist with autonomy to adjust the prescription medication; frequency of interventions more than once a month; and adequate random allocation (Table 2).

**Fig 3:**
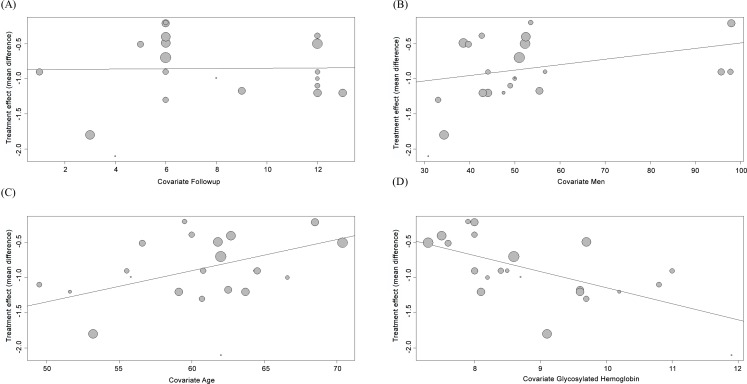
Meta-regression of the mean difference of HbA1c by (A) duration of follow-up; (B) baseline proportion of men; (C) baseline mean age and (D) baseline HbA1c levels.

The meta-regression indicated no association between the mean difference in HbA1c levels and duration of follow-up (Fig 3A; P = 0.952) or proportion of men among those RCTs examined (Fig 3B; P = 0.136). In contrast, we observed that mean differences in HbA1c levels decreased with increasing age (regression coefficient: 0.05 [95% IC: 0.01, 0.08]; p = 0.0085) (Fig 3C), and this variate explained 48.81% of the heterogeneity across studies (residual I^2^ = 50.1%; P = 0.0048). Finally, we found that mean differences in HbA1c levels increased with higher baseline HbA1c (regression coefficient: -0.23 [95% IC: -0.40, -0.06]; p = 0.0089) (Fig 3D), and this variate explained 32.47% of the heterogeneity in the effect on HbA1c (residual I^2^ = 57.5%; P = 0.0006).

### Sensitivity analysis

After excluding 12 low-quality studies (i.e., risk of bias score < 3 points using the Cochrane Risk of Bias Tool), we still noted statistically significant reductions in HbA1c levels with pharmacist intervention (mean difference for HbA1c of -1.05% [95% CI: -1.27, -0.82]; P < 0.0001), but with no heterogeneity across the RCTs. The next sensitivity analysis was limited to the 13 trials with sample size ≥100 patients, and similar reductions in HbA1c levels were observed in the intervention groups (-0.99% [95% CI: -1.25, -0.72]; P < 0.0001; I^2^ = 74.9%).

### Publication bias

No publication bias was detected in the current meta-analysis as can be seen by the symmetry of the funnel plot (S5 Appendix) and no significant result by the Egger’s test (t = -1.03, df = 20, p = 0.3171).

## Discussion

In consonance with two previous meta-analyses on this theme [10,11], our meta-analysis showed that pharmacist interventions have positive effects on reducing HbA1c levels in outpatients with type 2 diabetic. The detected effect (-0.85% in HbA1c levels) is notably relevant since that reductions in glycemic levels by 0.5% are associated with significant declines in diabetes-related endpoints and microvascular complications [49]. Added to this, a current systematic review [50] identified that pharmacist interventions are cost-saving because, although they increase medication costs, the medical costs (e.g. hospitalization and emergency department costs) are greatly decreased. Considering the escalating healthcare expenditures due to the inadequate diabetes management, the restructuring of health systems with expansion or revision of the pharmacist’s role can be an important strategy in addressing this challenge.

The included RCTs in this review presented high methodological and clinical heterogeneity, which produced substantial statistical heterogeneity in the effect estimate for HbA1c levels. Different geographical settings may have influenced this finding. Most studies were conducted in the United States, which confirms the pioneering role of this country on this practice [51] and may indicate a higher level of implementation of clinical pharmacist services. Thus, not surprisingly, all RCTs where the pharmacist had autonomy to change prescription medication were conducted in this country. Although these subgroups were not associated with better glycemic control in analyses, studies from the United States and studies whose pharmacist had autonomy to adjust the drug therapy showed greater homogeneity.

In our meta-analysis the effect size on HbA1c levels was inversely associated with age, probably because a less stringent glycemic control may be required for the elderly individual, depending on their health status [52]. In addition, we observed that pharmacist health interventions were more effective for patients with poor glycemic control, which is consistent with current meta-analyses on others intervention strategies for patients with diabetes [8,53]. The baseline characteristics of the participants may be potential sources of heterogeneity between trials [12]. Although the variables age and baseline HbA1c levels have explained part of the statistic heterogeneity, the residual heterogeneity remained equal to or above 50%.

The studies were principally conducted in outpatient clinics where pharmacists commonly worked collaboratively with the medical staff and discussed face-to-face on drug therapy problems. According to Van et al [54], the level of trust and cooperation of the physicians seems to be higher in this setting compared with a community pharmacy, where contacts are usually not personal and occur at a distance. Moreover, a pharmacist running a diabetes outpatient clinic may have a different level of knowledge, skills and experience in the condition and its management to a community pharmacist conducting a medication review [55]. Despite the trend towards greater reduction in HbA1c levels in a non-community pharmacy setting, trials vary widely in relation to pharmacist health interventions and population characteristics, generating results based on substantial statistical heterogeneity.

Among the key components of pharmacist interventions, the level of pharmacist-patient interaction presented great variability across studies. This finding may reflect the need to develop clinical pharmacy services adapted to the context reality, but also the uncertainty about which of the options could be more effective. Although increasing evidence indicates that telephone interventions are effective for glycemic control in patients with diabetes [56], our meta-analysis found no difference in effect compared with studies that performed face-to-face contact only. Furthermore, the duration of pharmacist intervention was not associated with change in HbA1c levels, contrary to a recent systematic review on patient activation interventions that showed the greatest improvements in HbA1c levels with a longer study duration [53].

Although most of the RCTs claimed to perform a medication review, there were poor descriptions of the methods used by the pharmacist, and among those that were described clearly, various aspects of the medication review process were assessed (drug selection or effectiveness or safety of medications). This may hinder understanding of the real power of medication review in subgroup analysis and lead to misinterpretation when comparing the results of different studies. In addition, a systematic review showed that the poor descriptions of pharmacist health interventions made the implementation of services developed for clinical practice difficult, which reinforces the need for clear and thorough reporting of these interventions [57].

All RCTs in this systematic review performed patient education that was similar about the content addressed, mainly in relation to diabetes complications, blood glucose monitoring and healthy lifestyle. However, there were differences or poor descriptions regarding the educational program structure and supporting materials offered to patients. Combined with health education delivered verbally, support material provides greater potential improvements in health outcomes [58,59]; despite that, the results of this meta-analysis showed similar effects and high heterogeneity with respect to the provision of supporting materials.

Although this study includes only RCTs, which are considered the gold standard for evidence-based health practice, methodological problems were recurrent, resulting in low-quality studies. Most RCTs did not clearly describe the allocation concealment, blinding, and reporting of selective outcome. On the other hand, the method of sequence generation was the item most usually reported. We note that whereas adequate random allocation or higher quality of the studies did not influence the effect of the pharmacist interventions on the reducing HbA1c levels, the heterogeneity of these studies was very low.

Finally, we believe that this systematic review constitutes a valuable addition to research and practice on pharmacist health interventions in patients with type 2 diabetes. Our findings provide insights into how and how much the pharmacist can contribute in reducing HbA1c levels. Potential sources of clinical, methodological, and statistical heterogeneity and complex intervention components that could influence the glycemic control (age, baseline HbA1c levels, and setting) were identified. In addition, our results point out the need for greater study design quality as well as a more detailed description of pharmacist interventions in future RCTs, so that meta-analysis studies can provide more robust results to guide the direction of pharmacist practice in the management of diabetes.

### Strengths and limitations

Our systematic review with meta-analysis made an active effort to overcome the identified gaps in an overview of pharmacist interventions for patients with diabetes [14], such as improving the quality of the evidence using PRISMA and AMSTAR tools and exploring heterogeneity by subgroup and meta-regression analyses. However, some limitations should be noted. As the literature search included only published articles, it is possible that studies with negative results were not caught; but it is worth mentioning that there was no observable publication bias in our meta-analysis. Secondly, the care received by the intervention and control groups was often poorly described, making it difficult to map the components of the pharmacist interventions and measure their actual effects. Finally, risk of bias in individual trials was poorly reported.

## Conclusions

Our findings confirmed that pharmacist interventions improve glycemic control in type 2 diabetes patients compared with usual care. Subgroup analyses showed greater homogeneity between RCTs conducted in the United States, with baseline HbA1c ≤ 9%, in community pharmacy setting, whose interventions are conducted at least monthly, and with adequate sequence generation; and covariates age and HbA1c levels partly explain the heterogeneity by meta-regression. In addition, our analyses suggest that younger patients or with higher baseline HbA1c levels may be the main beneficiaries of pharmacist care.

## Supporting Information

S1 Appendix PRISMA checklist. (DOCX)

S2 Appendix Full search strategy for RCT of pharmacist health interventions in PubMed/Medline. (DOCX)

S3 Appendix List of excluded articles. (DOCX)

S4 Appendix Risk of bias summary of included RCTs. (DOCX)

S5 Appendix Funnel plot for the mean difference of HbA1c levels. (DOCX)
