# Supplementary material for: Investigating Sources of Heterogeneity in Randomized Controlled Trials of the Effects of Pharmacist Interventions on Glycemic Control in Type 2 Diabetic Patients: A Systematic Review and Meta-Analysis
Source: PLoS One. 2016 Mar 10;11(3):e0150999. doi: 10.1371/journal.pone.0150999 (PMC4786227; doi:10.1371/journal.pone.0150999)
Supplement: S2 Appendix — (DOCX) [file pone.0150999.s002.docx]

**S2 Appendix: Full search strategy for RCT of pharmacist health interventions in PubMed/Medline.**

((“pharmaceutical care”[tw]) OR (“medication therapy management”[Mesh Terms]) OR (“pharmaceutical services”[Mesh Terms]) OR pharmacist*[tiab] OR pharmacists[MeSH Terms]) AND (“diabet*”[tiab] OR (“diabetes mellitus, type 2”[MeSH Terms]) OR (“blood glucose”[tw]) OR glycaemia[tiab] OR glycemi*[tiab] OR (“Hemoglobin A, glycosylated”[MeSH Terms]) OR (“glycosylated hemoglobin”[tw])) AND ((clinical[tiab] AND trial[tiab]) OR clinical trials as topic[Mesh Terms] OR clinical trial[pt] OR random*[tiab] OR random allocation[Mesh Terms] OR therapeutic use[Mesh subheading])
