# Supplementary material for: Investigating Sources of Heterogeneity in Randomized Controlled Trials of the Effects of Pharmacist Interventions on Glycemic Control in Type 2 Diabetic Patients: A Systematic Review and Meta-Analysis
Source: PLoS One. 2016 Mar 10;11(3):e0150999. doi: 10.1371/journal.pone.0150999 (PMC4786227; doi:10.1371/journal.pone.0150999)
Supplement: S3 Appendix — (DOCX) [file pone.0150999.s003.docx]

**S3 Appendix: List of excluded articles.**

| **Reason for exclusion** | **Authors, year** | **Title** | **Reference** |
| --- | --- | --- | --- |
| Not a RCT | Coast-Senior et al., 1998 | Management of patients with type 2 diabetes by pharmacists in primary care clinics. | Ann Pharmacother. 1998;32(6):636-41. |
|  | Davidson et al., 2000 | Effect of a pharmacist-managed diabetes care program in a free medical clinic. | Am J Med Qual. 2000;15(4):137-42. |
|  | Borges et al., 2010 | The pharmaceutical care of patients with type 2 diabetes mellitus. | Pharm World Sci. 2010;32(6):730-6. |
|  | Correr et al., 2011 | Effects of a pharmacotherapy follow-up in community pharmacies on type 2 diabetes patients in Brazil. | Int J Clin Pharm. 2011;33(2):273-80. |
|  | Mitchell et al., 2011 | Diabetes Medication Assistance Service: The pharmacist's role in supporting patient self-management of type 2 diabetes (T2DM) in Australia. | Patient Educ Couns. 2011;83(3):288-94. |
|  | Edwards et al., 2012 | A pharmacist visit improves diabetes standards in a patient-centered medical home (PCMH). | Am J Med Qual. 2012;27(6):529-34. |
| Patients with type 1 diabetes | Clifford et al, 2002 | A randomised controlled trial of a pharmaceutical care programme in high-risk diabetic patients in an outpatient clinic. | Int J Pharm Pract. 2002;10:85-9. |
|  | Phumipamorn et al., 2008 | Effects of the pharmacist’s input on glycaemic control and cardiovascular risks in Muslim diabetes. | Prim Care Diabetes. 2008;2(1):31-7. |
|  | Kraemer et al., 2012 | A Randomized Study to Assess the Impact of Pharmacist Counseling of Employer-Based Health Plan Beneficiaries With Diabetes: The EMPOWER Study. | J Pharm Pract. 2012;25(2):169-79. |
|  | Nishita et al., 2012 | Empowered Diabetes Management: Life Coaching and Pharmacist Counseling for Employed Adults With Diabetes. | Health Educ Behav. 2013;40(5):581-91. |
|  | Samtia et al., 2013 | A multifactorial intervention to enhance adherence to medications and disease-related knowledge in type 2 diabetic patients in Southern Punjab, Pakistan. | Trop J Pharm Res. 2013;12(5):851-6. |
| Various chronic diseases | Neto et al., 2011 | Effect of a 36-month pharmaceutical care program on coronary heart disease risk in elderly diabetic and hypertensive patients. | J Pharm Pharm Sci. 2011;14(2):249-63. |
|  | Obreli-Neto et al., 2011 | Effect of a 36-month pharmaceutical care program on pharmacotherapy adherence in elderly diabetic and hypertensive patients. | Int J Clin Pharm. 2011;33(4):642-9. |
|  | Molina-Lopez el al., 2012 | Revisión de la medicación en ancianos polimedicados en riesgo vascular: ensayo aleatorizado y controlado. | Aten Primaria. 2012;44(8):453-60. |
| Not evaluate HbA1c levels | Ladhani et al., 2012 | Adding pharmacists to primary care teams reduces predicted long-term risk of cardiovascular events in type 2 diabetic patients without established cardiovascular disease: results from a randomized trial. | Diabet Med. 2012;29(11):1433-9. |
|  | Planas et al., 2012 | Evaluation of a diabetes management program using selected HEDIS measures. | J Am Pharm Assoc. 2012;52(6):e130-8. |
| Not directed to the patient | Kirwin et al., 2010 | Pharmacist Recommendations to Improve the Quality of Diabetes Care: A Randomized Controlled Trial. | J Manag Care Pharm. 2010;16(2):104-13. |
| Non-ambulatory patients | Gangwar et al., 2014 | Impact of medication and psychological behaviour assessment by community pharmacists in type 2 diabetes mellitus patients after hospital stay. | Afr Health Sci. 2014;14(3):539-50. |
| Not focused on pharmacist intervention | O'Connor et al., 2014 | Randomized trial of telephone outreach to improve medication adherence and metabolic control in adults with diabetes. | Diabetes Care. 2014;37(12):3317-24. |
| Repeated study | Andrés Rodríguez et al., 2007 | Valoración del conocimiento/cumplimiento en un programa de seguimento farmacoterapéutico en diabéticos tipo 2 em farmacia comunitaria. | Pharm Care Esp. 2007;9(1):2-9. |
