# Supplementary figures and images for: Investigating Sources of Heterogeneity in Randomized Controlled Trials of the Effects of Pharmacist Interventions on Glycemic Control in Type 2 Diabetic Patients: A Systematic Review and Meta-Analysis
Source: PLoS One. 2016 Mar 10;11(3):e0150999. doi: 10.1371/journal.pone.0150999 (PMC4786227; doi:10.1371/journal.pone.0150999)

**S4 Appendix: Risk of bias summary of included RCTs.**


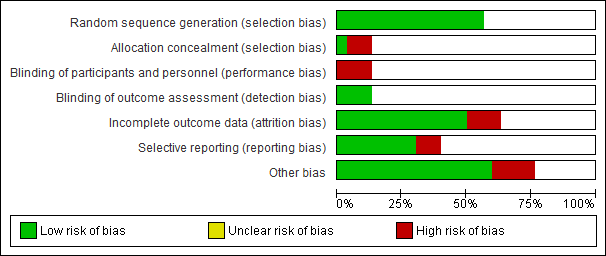

Supplement: S4 Appendix — (DOCX) [file pone.0150999.s004.docx]

**Appendix 5: Funnel plot for the mean difference of HbA1c levels.**


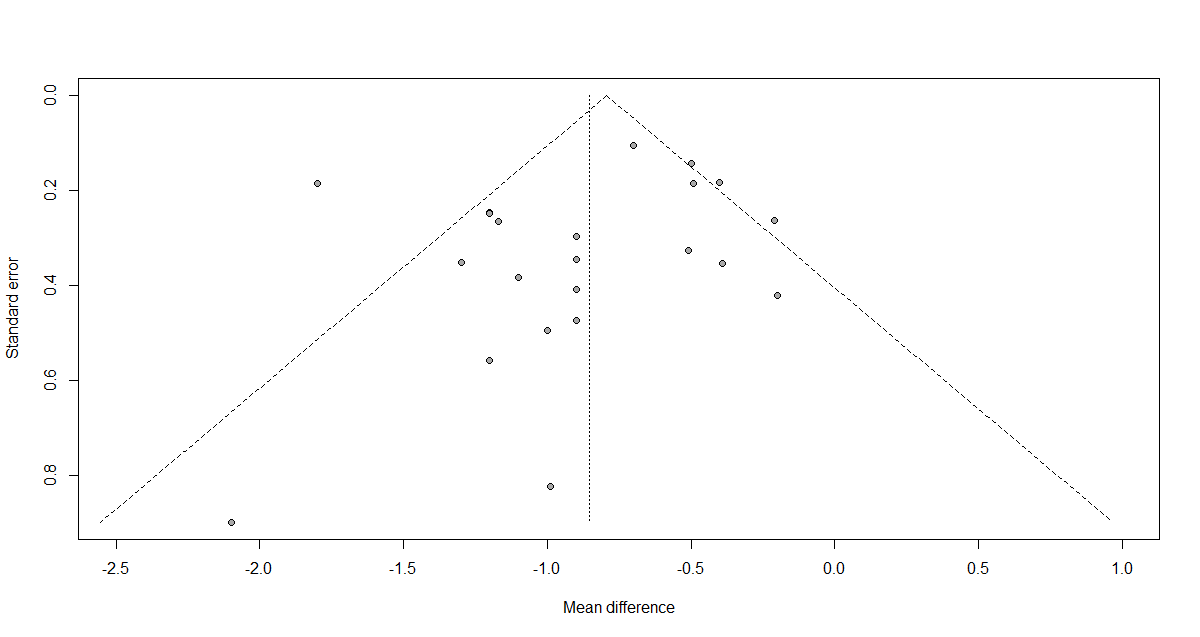

Supplement: S5 Appendix — (DOCX) [file pone.0150999.s005.docx]
